# Epidemiological profile of *Clonorchis sinensis* infection in one community, Guangdong, People’s Republic of China

**DOI:** 10.1186/1756-3305-6-194

**Published:** 2013-07-01

**Authors:** Men-Bao Qian, Ying-Dan Chen, Yue-Yi Fang, Tan Tan, Ting-Jun Zhu, Chang-Hai Zhou, Guo-Fei Wang, Long-Qi Xu, Xiao-Nong Zhou

**Affiliations:** 1 National Institute of Parasitic Diseases, Chinese Center for Disease Control and Prevention; WHO Collaborative Center for Malaria, Schistosomiasis and Filariasis; Key Laboratory of Parasite and Vector Biology, Ministry of Health, Shanghai, People’s Republic of China; 2 Center for Disease Control and Prevention of Guangdong Province, Guangzhou, People’s Republic of China; 3 Center for Disease Control and Prevention of Shunde District, Shunde, People’s Republic of China

**Keywords:** Epidemiology, *Clonorchis sinensis*, Infection, Food-borne parasitic diseases, Knowledge, Behavior

## Abstract

**Background:**

Clonorchiasis caused by ingesting improperly prepared fish ranks among the most important but still neglected food-borne parasitic diseases, especially in the People’s Republic of China (P.R. China). To promote the implementation of interventions efficiently, the demonstration of an epidemiological profile of *Clonorchis sinensis* infection is essential in hyper-epidemic areas.

**Methods:**

In one community with higher levels of economic development in Guangdong province, P.R. China, villagers were motivated to provide stool samples for examining helminth eggs. Then, those infected with *C*. *sinensis* completed the structured questionnaire including demographical characteristics, knowledge and behavior.

**Results:**

A total of 293 villagers infected with *C*. *sinensis* participated in questionnaire investigation. Among them, 94.54% were adult and 93.17% were indigenous. The geometric mean of *C*. *sinensis* eggs per gram of feces in the children, adult females and adult males was 58, 291 and 443, respectively. The divergence between knowledge and behavior in the adults, especially the adult males, was shown. Out of 228 persons eating raw fish, 160 did it more frequently at restaurants, the proportion of which varied in different populations, showing 25.00%, 54.88% and 80.28% in the children, adult females and adult males, respectively.

**Conclusions:**

Different interventions need to be adopted in different populations. Chemotherapy should be prioritized in the adults, especially the adult males. In addition, health education targeting the children, is essential and may play a crucial role in controlling clonorchiasis in the long term. In order to successfully control clonorchiasis, intervention in the restaurant should not be overlooked in some endemic areas.

## Background

Liver fluke infections are caused by ingestion of improperly prepared fish harboring infective metacercaria and lead to significant disease burden in East Asia [1-4]. *Clonorchis sinensis* infection is predominantly endemic in the People’s Republic of China (P.R. China), the Republic of Korea and northern Vietnam [5,6]. However, its importance in public health has been neglected by the international community for decades [5-8]. Fortunately, changes occur gradually, for instance, liver fluke infections are included in the disease burden evaluation for food-borne diseases and the first report on neglected tropical diseases by WHO [8,9]. As for clonorchiasis, comprehensive intervention strategies based on chemotherapy have been implemented in two endemic counties in P.R. China since 2006 [10]. Chemotherapy, as the core of intervention in controlling clonorchiasis, demonstrated promising outcomes in pilot studies [10-12]. However, the sustainability of achievements in the long run is challenging, as re-infection cannot be avoided in chemotherapy, especially in the older age groups [8,13]. Health education is considered to be an important measurement, but no objective assessment is available [13]. Furthermore, unlike other parasitic diseases, clonorchiasis is endemic both in underdeveloped and developed areas [5,6,14-16]. Thus, capturing the epidemiological profiles in different endemic circumstances will promote the intervention effectively and sustainably [17,18]. The prevalence of clonorchiasis in Guangdong province ranks the top in P.R. China, especially in the developed Pearl Delta [14,15,19,20]. In this study, the epidemiological profile of *C*. *sinensis* infection in one community located in the Pearl Delta is presented.

## Methods

### Study site

The study was carried out in one community in Shunde district, Guangdong province, P.R. China. According to recent reports, clonorchiasis is hyper-epidemic in Shunde district [20], but the clear epidemiological picture in this community is not available. The per capita annual net income reached 11 800 RMB (about 1 815 dollars) in 2010 in this community.

### Fecal examination

One stool sample was collected from each participant. Triple Kato-Katz thick smears were prepared for each sample, and then examined under a light microscope to distinguish and count eggs [21]. The number of eggs per gram of feces (EPG) was calculated through multiplying the egg count of every smear by 24 and then computing the average of three smears.

### Questionnaire survey

After fecal examination, participants confirmed as infected with *C*. *sinensis* other than any other helminth infection were asked to complete the structured questionnaire by trained investigators. The questionnaire contained three parts, i.e. demographical characteristics, knowledge and behavior related to *C*. *sinensis* infection. In the demographical part, habitation history, as well as sex and age were recorded. Three questions related to knowledge and another four involving behavior were explored. During a pilot interview in a neighboring community, it was found that both raw and undercooked fish is enjoyed by local people. Ingesting undercooked fish is called “*dabianlu*” locally, namely eating the flesh after blanched in hot water for a few seconds or minutes. “*Dabianlu*” occurs predominantly at home, while eating raw fish called “*yusheng*” locally occurs both at home and restaurants. Thus, another question regarding where eating “*yusheng*” occured was included for those individuals eating raw fish. Detailed information on the questionnaire is listed in Table 1.

**Table 1:** Content and options of the structured questionnaire

**Items**	**Options**
1 Basic information	
1.1 Sex	Male = 1, Female = 2
1.2 Age	Years old
1.3 Indigenous	Yes = 1, No = 0
2 Knowledge	
2.1 ^#^Do you hear of clonorchiasis?	Yes = 1, No = 0
2.2 Do you know the transmission route of clonorchiasis?	Yes = 1, No = 0
2.3 Do you know the harm of clonorchiasis?	Yes = 1, No = 0
3 Behavior	
3.1 ^*^Do you eat raw fish (“*yusheng*”)?	0 time per year = 0, 1–4 times per year = 1, 5 times or above per year = 2
3.2 If you eat raw fish, where do you eat more frequently, at restaurants or home?	At restaurants = 1, At home = 2
3.3 Do you eat undercooked fish (“*dabianlu*”)?	0 time per year = 0, 1–4 times per year = 1, 5 times or above per year = 2
3.4 Is cooked and uncooked food prepared separately at home?	Yes = 1, No = 0

Note: ^#^ Only after obtaining the answer "Yes" in question 2.1, questions 2.2 and 2.3 will be asked continually.

^*^ Only after obtaining the answer "Yes" in question 3.1, question 3.2 will be asked continually.

### Data analysis

Data were double-entered and cross-checked in EpiDate3.1 software (http://www.epidata.dk/). Analysis was run in SPSS for Windows (version11.0; SPSS Institute, Inc., Chicago, IL). Due to the deviation from normality distribution, infection intensity in terms of EPG was transformed into logarithm, namely Lg(EPG). Student’s t test or analysis of variance (ANOVA) was employed for comparison among different groups, and then least significant difference (LSD) test if necessary was adopted for comparison within groups. Age was transformed into two categories, i.e. the children (less than 14 years old) and the adults (more than 15 years old). Pearson *x*^2^ test or Fisher’s exact test, when appropriate was applied to assess the association between category variables. Statistical significance was given at a *p*-value of 0.05. To obtain geometric means of EPG (GMEPG), the average of Lg(EPG) was calculated and then inversely logarithmically transformed.

### Ethical statement

The study was embedded in another study for evaluating the disease burden of *C*. *sinensis* infection, which was approved by the ethics committee in the National Institute of Parasitic Diseases, Chinese Center for Disease Control and Prevention (Ref No: 20100525–1). The objectives, procedures and potential risks were orally explained and informed to all participants. A written consent form was also obtained from each participant with signature of him or his proxy. After the study, those infected with *C*. *sinensis* and (or) soil-transmitted helminthes were treated free of charge.

## Results

### Infection status

A total of 1 385 villagers participated in fecal examination. 510 persons were infected with *C*. *sinensis*, out of which 5 individuals were co-infected with soil-transmitted helminthes. Finally, 293 persons with *C*. *sinensis* mono-infection accepted questionnaire investigation.

### Basic characteristics

Basic characteristics of 293 participants are summarized in Table 2. There were 165 males and 128 females. Age ranged from 7 to 77, with the mean and median of 45 and 46, respectively. Most were adults, accounting for 94.54%. There were 20 immigrants, who had inhabited in the region for more than 1 year. The percentage of individuals who had heard of clonorchiasis, knowing the transmission route and being aware of the harm was 72.35%, 64.51% and 44.71%, respectively, while that of knowing both the transmission route and harm reached 44.03%. The percentage of eating raw fish 0 time per year, 1–4 times per year and 5 times or above per year was 22.18%, 37.54% and 40.27%, respectively. However, 92.15% of persons ate undercooked fish 5 times or above per year. About 70.18% of responders reported they ate raw fish more frequently at restaurants. Only 37 participants (12.63%) reported that cooked and uncooked food was prepared separately at home.

**Table 2:** **Basic characteristics of 293 participants with *C***. ***sinensis *infection in this study**

**Items**	**Count**	**Total**	**Percentage (%)**
Sex			
Male	165	293	56.31
Female	128	293	43.69
Age			
The children	16	293	5.46
The adults	277	293	94.54
Indigenous			
Yes	273	293	93.17
No	20	293	6.83
Hearing of clonorchiasis			
Yes	212	293	72.35
No	81	293	27.65
Knowing the transmission route of clonorchiasis			
Yes	189	293	64.51
No	104	293	35.49
Knowing the harm of clonorchiasis			
Yes	131	293	44.71
No	162	293	55.29
Knowing the transmission route and harm of clonorchiasis^#^			
Yes	129	293	44.03
No	164	293	55.97
Eating raw fish			
0 time per year	65	293	22.18
1-4 times per year	110	293	37.54
5 times or above per year	118	293	40.27
Place where eating raw fish occurred more frequently			
At restaurants	160	228	70.18
At home	68	228	29.82
Eating undercooked fish			
0 time per year	1	293	0.34
1-4 times per year	22	293	7.51
5 times or above per year	270	293	92.15
Preparing cooked and uncooked food separately at home.			
Yes	37	293	12.63
No	256	293	87.37

Note: ^#^ The variable is newly created based on the two questions above, namely knowing the transmission route and knowing the harm.

### Infection intensity among different groups

Due to the small sample size of the children (only 8 boys and 8 girls) and the insignificant difference of infection intensity between them (t = −1.306, *p* = 0.213), the children of different sexes were combined into a single category. The GMEPG in the children, adult females and adult males was 58, 291 and 443, respectively (F = 12.237, *p* < 0.001). The difference was significant within groups (*p* < 0.001 in the children *vs* the adult females or the adult males; *p* = 0.033 in the adult females *vs* the adult males).

### Knowledge among different groups

**Figure 1:**
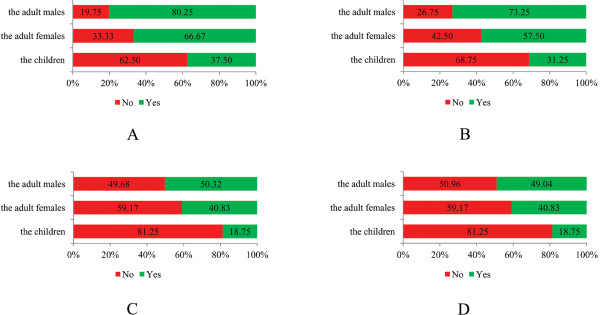
**Variance of knowledge in different populations. A**: Knowledge on hearing of clonorchiasis. **B**: Knowledge on knowing the transmission route of clonorchiasis. **C**: Knowledge on knowing the harm of clonorchiasis. **D**: Knowledge on knowing both the transmission route and harm of clonorchiasis.

Because of the insignificant difference of knowledge between the boys and girls (Fisher’s exact test, *p* = 1.000, in all items on knowledge), they were combined again here. The difference of having heard of clonorchiasis was significant among the children, adult females and adult males (*x*^2^ =16.557, *p* < 0.001; Figure 1A) and so was the difference in knowledge of the transmission route (*x*^2^ =15.542, *p* < 0.001; Figure 1B), knowledge about the harm (*x*^2^ =7.089, *p* = 0.029; Figure 1C) and knowing both the transmission route and harm (*x*^2^ =6.249, *p* = 0.044; Figure 1D).

### Behavior among different groups

**Figure 2:**
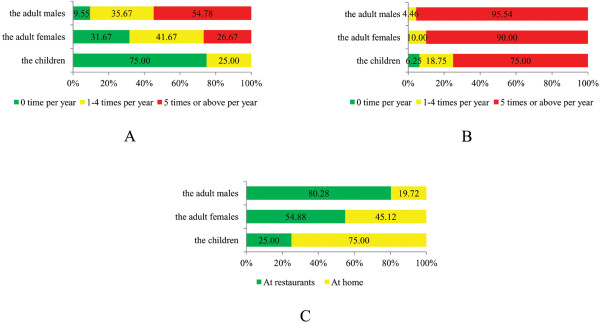
**Variances of behavior among different populations. A**: Eating raw fish. **B**: Eating undercooked fish. **C**: The place where eating raw fish occurred more frequently.

Similarly, there was no significant difference in behavior between the boys and girls (*p* = 1.000 in eating raw fish and *p* = 0.567 in eating undercooked fish), therefore, they were also combined here. The behavior of eating raw fish was differential among the children, adult females and adult males (*x*^2^ =57.794, *p* < 0.001; Figure 2A) and so was the behavior of eating undercooked fish (Fisher’s exact test, *p* = 0.005; Figure 2B). It was also significantly different among different groups where eating raw fish occurred more frequently (Fisher’s exact test, *p* < 0.001; Figure 2C).

### Relationship between the infection intensity and eating raw fish

To increase the comparability, the relationship between the infection intensity and eating raw fish in 270 persons consuming undercooked fish 5 times or above per year was explored. The number of persons eating raw fish 0 time, 1–4 times and 5 times or above per year was 53, 101 and 116, respectively, and corresponding GMEPG was 124, 249 and 736, respectively. The difference was significant (F = 28.079, *p* < 0.001; *p* = 0.008, 1–4 times *vs* 0 time; *p* < 0.001, 5 times or above *vs* 0 time or 1–4 times).

## Discussion

Responses on how to control and eventually eliminate human helminthiases require sound research to improve current tools and strategies [22]. Social ecology is just one indispensable aspect [23]. It is argued that the limited success of the numerous campaigns on controlling fish-borne zoonotic trematodiasis in some areas was due to the fact that such campaigns were not built on insights into the knowledge, practices and attitudes of people [24]. Thus, strengthening research on clonorchiasis is important, especially in P.R. China where accounting for over half of the population with liver fluke infections globally [5,6,17]. Here, the epidemiological profile of *C*. *sinensis* infection in one community where there is a highly developed economy with an annual net income over 1500$ per capital in Guangdong province was presented, aiming at provoking some inspirations for future interventions.

First of all, the divergence between knowledge and behavior, as well as infection intensity in the children, adult females and adult males indicates that different targets and interventions should be adopted in different populations. On the one hand, the adults, especially the adult males have more improper behavior and infection intensity, causing higher disease burden [25]. On the other hand, they have already had some preliminary knowledge, challenging the effect of conventional health education. Raw-fish-eating behavior is deeply rooted and difficult to change in this population [8,26]. Thus, lowering infection intensity and subsequent morbidity are most important and urgent for them. Obviously, chemotherapy will undoubtedly play a predominant role. Distinctly, due to the lower level of improper behavior and infection intensity as well as knowledge in the children, health education should be launched to foster health behavior, which will benefit the control of clonorchiasis in the long term. Of course, the discrepancy between knowledge and behavior in parents will impact negatively on their children. In particular, the situation will be made worse if parents encourage their children to eat raw fish due to traditional ideas that raw fish can make their children strong in body [27,28].

Secondly, among the infected, 6.83% are immigrants. Although the prevalence is not available here, it indicates the immigrants should not be neglected during the control of clonorchiasis. Owing to the rapid economic development, more and more immigrants from less developed areas are working and living in Guangdong province, especially in the developed area of the Pearl Delta. According to national censuses, the population in Guangdong province increased from 86.42 million in 2000 to 104.30 million in 2010 [29,30], and the proportion in the national population also increased from 6.83% to 7.79% [29]. The number of immigrants from other provinces reached 21.50 million in 2010, an increase of 42.71% compared to that in 2000. Additionally, the movement of the population also occurred within Guangdong province, which reached 9.78 million in 2010, with an increase rate of 63.34% compared to that in 2000 [30]. The immigrants mainly flow to more developed areas of the Pearl Delta that is a hyper-epidemic focus of clonorchiasis [19,20]. Because of assimilation by local people, the immigrants may get accustomed to the habit of raw-fish-eating gradually. On account of controlling infection source and social equality, the immigrants should also be paid enough attention and be included in the future control and prevention programs.

Thirdly, the styles of ingesting improperly prepared fish varied, such as fermented, roasted and even raw [14,31,32]. In this community, both raw (“*yusheng*”) and undercooked fish (“*dabianlu*”) is enjoyed by local people. Generally, eating “*yusheng*” is more dangerous than “*dabianlu*”, because *C*. *sinensis* metacercaria in the former are still in infectious status, while those in the latter may be dead. Whether metacercaria survive in “*dabianlu*” depends on the thickness of each piece of sliced flesh, the time blanched in water and the temperature of the water. It was found that only one person had not eaten “*dabianlu*” but up to 22% had not ingested “*yusheng*”, which means eating “*dabianlu*” is one important infection route. Additionally, only 12.83% of persons reported that the cooked and uncooked food is prepared separately at home. Thus, food contamination may also be another important transmission route.

Fourthly, among those eating raw fish, up to 70% of people do it more frequently at restaurants, with significant difference among different populations. Although raw fish needn’t be cooked, the preparation is not simple. For example, the fish should be cut into slices as thin as possible without blood and many additional dishes should be provided. Thus, raw fish made at restaurants is preferred. Furthermore, high economic development has made it possible for more people to be able to afford to go to restaurants. Traditionally, household hygiene is considered to be important in the control of parasite infections [33,34]. Although household hygiene is still necessary in controlling clonorchiasis, the improperly preparing food or fish at restaurants is important factor in the transmission of clonorchiasis. How to integrate restaurant hygiene into the control program is another issue that needs to be dealing with.

Three limitations exist in this study. Firstly, apparently, no control group was included. Therefore, many indexes compare constituent ratio, other than incidence or prevalence. However, this study focuses on the epidemiological profile of infected populations. Through classifying the infected population into different groups and then analyzing and comparing the corresponding characteristics, some aspirations for interventions are well presented. Secondly, the household status is not elucidated. Therefore, it is not clear how the mutual impact occurs among members within individual families, which needs to be explored in further studies. Thirdly, the economic and social developments of the epidemic areas of clonorchiasis vary markedly. Thus, the findings here may not represent the characteristics in other less developed areas and related surveys in other environmental settings are expected. In a word, further studies in social ecology will promote the control and even final elimination of clonorchiasis in P.R. China [35,36].

## Conclusions

Difference and even discrepancy in knowledge, behavior and infection intensity existed among different populations. Thus, different intervention measurements need to be adopted in different populations. Chemotherapy should be prioritized in the adults, especially the adult males. Health education targeting the children is essential and may play a crucial role in control of clonorchiasis in the long term. Immigrants are necessary to be paid attention and to be included in the future control programs. In order to successfully control clonorchiasis, intervention in restaurants should not be overlooked in some endemic areas.

## Competing interests

The authors declare that they have no competing interests.

## Authors’ contributions

Conceived and designed the experiments: MBQ YDC YYF LQX XNZ. Performed the experiments: MBQ YDC YYF TT TJZ CHZ GFW. Analyzed the data: MBQ. Contributed reagents/materials/analysis tools: MBQ YDC YYF. Wrote the paper: MBQ XNZ. All authors read and approved the final version of the manuscript.
